# ACPA-Negative RA Consists of Two Genetically Distinct Subsets Based on RF Positivity in Japanese

**DOI:** 10.1371/journal.pone.0040067

**Published:** 2012-07-06

**Authors:** Chikashi Terao, Koichiro Ohmura, Katsunori Ikari, Yuta Kochi, Etsuko Maruya, Masaki Katayama, Kimiko Yurugi, Kota Shimada, Akira Murasawa, Shigeru Honjo, Kiyoshi Takasugi, Keitaro Matsuo, Kazuo Tajima, Akari Suzuki, Kazuhiko Yamamoto, Shigeki Momohara, Hisashi Yamanaka, Ryo Yamada, Hiroo Saji, Fumihiko Matsuda, Tsuneyo Mimori

**Affiliations:** 1 Department of Rheumatology and Clinical Immunology, Kyoto University Graduate School of Medicine, Kyoto, Japan; 2 Center for Genomic Medicine, Kyoto University Graduate School of Medicine, Kyoto, Japan; 3 Institute of Rheumatology, Tokyo Women’s Medical University, Tokyo, Japan; 4 Laboratory for Autoimmune Diseases, Center for Genomic Medicine, RIKEN, Yokohama, Japan; 5 HLA Laboratory, Kyoto, Japan; 6 Department of Transfusion Medicine and Cell Therapy, Kyoto University Hospital, Kyoto, Japan; 7 Department of Rheumatology, Sagamihara National Hospital, National Hospital Organization, Sagamihara, Japan; 8 Department of Rheumatology, Niigata Rheumatic Center, Niigata, Japan; 9 Rheumatoid Arthritis Center, Saiseikai Takaoka Hospital, Toyama, Japan; 10 Department of Internal Medicine, Center for Rheumatic Diseases, Dohgo Spa Hospital, Matsuyama, Japan; 11 Aichi Cancer Center Hospital and Research Institute, Nagoya, Japan; 12 Department of Allergy and Rheumatology, Graduate School of Medicine, University of Tokyo, Tokyo, Japan; 13 CREST program, Japan Science and Technology Agency, Kawaguchi, Saitama, Japan; 14 Institut National de la Sante et de la Recherche Medicale (INSERM) Unite U852, Kyoto University Graduate School of Medicine, Kyoto, Japan; Institut Jacques Monod, France

## Abstract

HLA-DRB1, especially the shared epitope (SE), is strongly associated with rheumatoid arthritis (RA). However, recent studies have shown that SE is at most weakly associated with RA without anti-citrullinated peptide/protein antibody (ACPA). We have recently reported that ACPA-negative RA is associated with specific HLA-DRB1 alleles and diplotypes. Here, we attempted to detect genetically different subsets of ACPA-negative RA by classifying ACPA-negative RA patients into two groups based on their positivity for rheumatoid factor (RF). HLA-DRB1 genotyping data for totally 954 ACPA-negative RA patients and 2,008 healthy individuals in two independent sets were used. HLA-DRB1 allele and diplotype frequencies were compared among the ACPA-negative RF-positive RA patients, ACPA-negative RF-negative RA patients, and controls in each set. Combined results were also analyzed. A similar analysis was performed in 685 ACPA-positive RA patients classified according to their RF positivity. As a result, HLA-DRB1*04:05 and *09:01 showed strong associations with ACPA-negative RF-positive RA in the combined analysis (p = 8.8×10^−6^ and 0.0011, OR: 1.57 (1.28–1.91) and 1.37 (1.13–1.65), respectively). We also found that HLA-DR14 and the HLA-DR8 homozygote were associated with ACPA-negative RF-negative RA (p = 0.00022 and 0.00013, OR: 1.52 (1.21–1.89) and 3.08 (1.68–5.64), respectively). These association tendencies were found in each set. On the contrary, we could not detect any significant differences between ACPA-positive RA subsets. As a conclusion, ACPA-negative RA includes two genetically distinct subsets according to RF positivity in Japan, which display different associations with HLA-DRB1. ACPA-negative RF-positive RA is strongly associated with HLA-DRB1*04:05 and *09:01. ACPA-negative RF-negative RA is associated with DR14 and the HLA-DR8 homozygote.

## Introduction

Rheumatoid arthritis (RA) is the most common cause of chronic arthritis worldwide and results in severe joint destruction [1]. Genetic and environmental factors have been shown to be associated with its onset [2]–[3]. Among the susceptibility genes to RA, HLA-DRB1 has been shown to be the strongest genetic determinant of RA susceptibility, and its association with RA susceptibility has been repeatedly shown to be independent of ethnicity [4]–[5]. A common amino acid sequence extending from the 70^th^ to 74^th^ in the HLA-DRβ chain, which is known as the “shared epitope (SE)”, is considered to be the reason for the association between HLA-DRB1 and RA, and the association between the SE and RA has been reported to be ethnicity-independent [6]–[8]. However, recent studies have shown that the SE is strongly associated with RA patients who have anti-citrullinated peptide/protein antibodies (ACPA), which is a highly specific marker of RA [9], but that it is not or only weakly associated with RA without ACPA [7], [10]–[11]. Among the various HLA-DRB1 alleles, HLA-DR3 [12] and HLA-DR13 [13] were reported to be associated with ACPA-negative RA in populations of European descent, but these results were not confirmed in a meta-analysis of a large Caucasian cohort [8]. In Asian populations, we recently reported that DRB1*12:01 is a HLA-DRB1 susceptibility allele for ACPA-negative RA in Japanese populations and that DRB1*04:05, the most common SE allele in Japanese, and *14:03 showed moderate associations with ACPA-negative RA susceptibility [14]. We also reported that DRB1*15:02 and *13:02 displayed protective associations with ACPA-negative RA and that being homozygous for HLA-DR8 was associated with ACPA-negative RA susceptibility. While a very small Japanese study suggested that HLA-DRB1*09:01 is associated with ACPA-negative RA [15], our study did not detect a significant association between them. These findings suggest that ACPA-negative RA is genetically different from ACPA-positive RA in terms of its associations with HLA-DRB1 alleles. While some specific alleles and diplotypes seem to be associated with ACPA-negative RA, the genetic characteristics of ACPA-negative RA have not been fully elucidated. Recently, UK group reported that SE is associated with ACPA-negative RF-positive RA in UK population [16]. However, whether this is true to other population is uncertain. Moreover, the associations of other alleles than SE with subgroups of ACPA-negative RA have never been reported. Here, we show that when we classified ACPA-negative RA into two subsets based on rheumatoid factor (RF) positivity, we were able to clearly distinguish them from each other according to their associations with HLA-DRB1 alleles, not only with SE, but with other alleles. We also compared ACPA-positive RA patients based on their RF positivity to examine whether we can apply this classification to ACPA-positive RA.

## Results

### HLA-DRB1 Alleles Associated with ACPA-negative RF-positive RA

We compared 179 ACPA-negative RF-positive RA with 1508 controls in collection 1 for their frequency of HLA-DRB1 alleles, followed by comparison of 267 ACPA-negative RF-positive RA with 500 controls in collection 2. Significant association was evaluated in the combined analysis. Regarding HLA-DRB1 alleles that were previously shown to be associated with ACPA-negative RA, we found that all of the alleles, namely, HLA-DRB1*12:01, *04:05, *13:02, *14:03, and *15:02 showed association tendency with ACPA-negative RF-positive RA in the combined study (Table 1). Interestingly, HLA-DRB1*04:05 (p = 8.8×10^−6^, odds ratio (OR): 1.57) showed the strongest association, while its association with entire ACPA-negative RA was moderate in the previous study. When we analyzed the associations of the SE, we found that it displayed a significant association (p = 0.00013, OR: 1.37). HLA-DRB1*04:05 was responsible for most of the association of SE because none of the other SE alleles showed significant associations with ACPA-negative RF-positive RA. We also found that HLA-DRB1*09:01, which was not associated with ACPA-negative RA as a single allele, was found to be significantly associated with ACPA-negative RF-positive RA (p = 0.0011, OR: 1.37). Importantly, these association tendencies written above were observed in both collections (Table 1). Logistic regression analysis was carried out to examine whether the susceptibility associations were dependent on a lack of protective alleles or vice versa. As a result, it was demonstrated that HLA-DRB1*04:05, *09:01, and *12:01 showed significant associations (p<0.0005), while the associations of HLA-DRB1*14:03, *13:02, and *15:02 were moderate to suggestive (Table S1). Next, we analyzed the dosage effects of the alleles and found that the association between HLA-DRB1*09:01 and ACPA-negative RF-positive RA showed a clear dosage effect (Figure S1). HLA-DRB1*12:01 also showed a dosage effect (data not shown due to small number). HLA-DRB1*04:05 did not show a dosage effect, suggesting that the effect of HLA-DRB1*04:05 on the predisposition to ACPA-negative RF-positive RA is a dominant effect.

**Table 1:** Association of HLA-DRB1 alleles with ACPA-negative RF-positive RA.

	1st set				2nd set				combined analysis			
HLA-DRB1 allele	^§^ACPA (-)RF(+)RA	^§^control	*p*	OR	^§^ACPA (-)RF(+)RA	^§^control	*p*	OR	^§^ACPA (-)RF(+)RA	^§^control	*p*	OR
*04:05	65 (18.2%)	340 (11.3%)	0.00015	1.75 (1.30–2.34)	88 (16.5%)	129 (12.9%)	0.055	1.33 (0.99–1.79)	153 (17.2%)	469 (11.7%)	8.8×10^−6^	1.57 (1.28–1.91)
*09:01	70 (19.6%)	432 (14.3%)	0.0086	1.45 (1.10–1.92)	99 (18.5%)	154 (15.4%)	0.11	1.25 (0.95–1.65)	169 (18.9%)	586 (14.6%)	0.0011	1.37 (1.13–1.65)
*12:01	13 (3.6%)	91 (3%)	0.53	1.21 (0.67–2.19)	35 (6.6%)	37 (3.7%)	0.012	1.83 (1.14–2.93)	48 (5.4%)	128 (3.2%)	0.0014	1.73 (1.23–2.43)
*13:02	21 (5.9%)	273 (9.1%)	0.043	0.63 (0.40–0.99)	18 (3.4%)	52 (5.2%)	0.10	0.64 (0.37–1.1)	39 (4.4%)	325 (8.1%)	0.00013	0.52 (0.37–0.73)
*14:03	7 (2.0%)	39 (1.3%)	0.31	1.52 (0.68–3.43)	13 (2.4%)	14 (1.4%)	0.14	1.76 (0.82–3.77)	20 (2.2%)	53 (1.3%)	0.040	1.71 (1.02–2.88)
*15:02	43 (12.0%)	369 (12.2%)	0.90	0.98 (0.70–1.37)	37 (6.9%)	113 (11.3%)	0.0060	0.58 (0.4–0.86)	80 (9.0%)	482 (12.0%)	0.010	0.72 (0.56–0.93)
SE	106 (29.6%)	677 (22.4%)	0.0024	1.45 (1.14–1.85)	150 (28.1%)	233 (23.3%)	0.039	1.29 (1.01–1.63)	256 (28.7%)	910 (22.7%)	0.00013	1.37 (1.17–1.62)
DR14	29 (8.1%)	253 (8.4%)	0.85	0.96 (0.64–1.44)	48 (9.0%)	73 (7.3%)	0.24	1.25 (0.86–1.83)	78 (8.7%)	326 (8.1%)	0.55	1.08 (0.83–1.40)
Diplotype												
DR8/DR8	3 (1.7%)	17 (1.1%)	0.46	1.49 (0.28–5.24)	3 (1.1%)	8 (1.6%)	0.76	0.70 (0.12–2.94)	6 (1.3%)	25 (1.2%)	0.86	1.08 (0.44–2.65)
*12:01/*09:01	5 (2.8%)	10 (0.66%)	0.0041	4.30 (1.45–12.74)	9 (3.3%)	3 (0.60%)	0.0051	5.76 (1.42–33.42)	14 (3.1%)	13 (0.6%)	5.0×10^−6^	4.97 (2.32–10.66)

OR: odds ratio.

SE: shared epitope: HLA-DRB1*01:01, *01:02, *04:01, *04:04, *04:05, *04:08, *04:10, *04:13, *04:16, *10:01, *14:02, and *14:06.

### HLA-DRB1 Alleles Associated with ACPA-negative RF-negative RA

Next we compared 274 ACPA-negative RF-negative RA with 1,508 controls, followed by comparison between 234 ACPA-negative RF-negative RA and 500 controls. Interestingly, we did not observe association of HLA-DRB1*04:05 and *09:01 with ACPA-negative RF-negative RA, while HLA-DRB1*12:01, *13:02, *14:03, and *15:02 were moderately associated with ACPA-negative RF-negative RA (Table 2). The SE was not associated with ACPA-negative RF-negative RA. DR14 was found to be significantly associated with ACPA-negative RF-negative RA and HLA-DRB1*14:03 and *14:06 comprised the association of HLA-DR14 (Table S2). These association tendencies in ACPA-negative RF-negative RA were observed in both sets (Table 2). Logistic regression analysis confirmed that none of the associations were mutually dependent and that the association of DR14 remained significant (p = 0.00069, Table S3). DR14 could not be evaluated the dosage effect because neither the cases nor controls included DRB1*14:03 or *14:06 homozygotes or the DRB1*14:03 and *14:06 diplotype.

**Table 2:** Association of HLA-DRB1 alleles with ACPA-negative RF-negative RA.

	1st set				2nd set				combined analysis			
HLA-DRB1 allele	^§^ACPA(-)RF(-)RA	^§^control	*p*	OR	^§^ACPA(-)RF(-)RA	^§^control	*p*	OR	^§^ACPA(-)RF(-)RA	^§^control	*P*	OR
*04:05	69 (12.6%)	340 (11.3%)	0.37	1.13 (0.86–1.49)	57 (12.2%)	129 (12.9%)	0.70	0.94 (0.67–1.31)	126 (12.4%)	469 (11.7%)	0.52	1.07 (0.87–1.32)
*09:01	74 (13.5%)	432 (14.3%)	0.61	0.93 (0.72–1.22)	65 (13.9%)	154 (15.4%)	0.45	0.89 (0.65–1.21)	139 (13.7%)	586 (14.6%)	0.46	0.93 (0.76–1.13)
*12:01	28 (5.1%)	91 (3.0%)	0.012	1.73 (1.12–2.67)	27 (5.8%)	37 (3.7%)	0.070	1.59 (0.96–2.65)	55 (5.4%)	128 (3.2%)	0.00071	1.74 (1.26–2.40)
*13:02	28 (5.1%)	273 (9.1%)	0.0023	0.54 (0.36–0.81)	34 (7.3%)	52 (5.2%)	0.070	1.59 (0.96–2.65)	62 (6.1%)	325 (8.1%)	0.033	0.74 (0.56–0.98)
*14:03	12 (2.2%)	39 (1.3%)	0.10	1.71 (0.89–3.29)	10 (2.1%)	14 (1.4%)	0.30	1.54 (0.68–3.49)	22 (2.2%)	53 (1.3%)	0.047	1.65 (1.00–2.73)
*15:02	51 (9.3%)	369 (12.2%)	0.051	0.74 (0.54–1.00)	36 (7.7%)	113 (11.3%)	0.033	0.65 (0.44–0.97)	87 (8.6%)	482 (12.0%)	0.0020	0.69 (0.54–0.87)
SE	131 (23.9%)	677 (22.4%)	0.45	1.09 (0.88–1.34)	103 (22%)	233 (23.3%)	0.58	0.93 (0.71–1.21)	234 (23.0%)	910 (22.7%)	0.80	1.02 (0.87–1.2)
DR14	69 (12.6%)	253 (8.4%)	0.0016	1.57 (1.19–2.09)	51 (10.9%)	73 (7.3%)	0.021	1.55 (1.07–2.26)	120 (11.8%)	326 (8.1%)	0.00022	1.52 (1.21–1.89)
Diplotype												
DR8/DR8	12 (4.4%)	17 (1.1%)	9.1×10^−5^	4.02 (1.90–8.51)	7 (3.0%)	8 (1.6%)	0.21	1.90 (0.68–5.29)	19 (3.7%)	25 (1.2%)	0.00013	3.08 (1.68–5.64)
*12:01/*09:01	4 (1.5%)	10 (0.66%)	0.25	2.22 (0.50–7.76)	4 (1.7%)	3 (0.60%)	0.22	2.88 (0.48–19.80)	8 (1.6%)	13 (0.6%)	0.040	2.46 (1.01–5.96)

### HLA Diplotype Analysis: DR8 Homozygote and *12:01/*09:01 Diplotype

As we previously showed that the DR8 homozygote was significantly associated with susceptibility to ACPA-negative RA, we analyzed its associations with ACPA-negative RF-positive RA and RF-negative RA. As a result, we found that the HLA-DR8 homozygote is exclusively associated with ACPA-negative RF-negative RA in the combined study (p = 0.00013, OR: 3.08 for ACPA-negative RF-negative RA, Table 2; p = 0.86, OR: 1.08 for ACPA-negative RF-positive RA, Table 1). The effect of DR8 on the susceptibility to ACPA-negative RF-negative RA was not dose-dependent (OR: 1.04 for HLA-DR8 heterozygote).

We also found that the combination of HLA-DRB1*12:01 and *09:01, the diplotype that was most strongly associated with susceptibility to ACPA-negative RA in the previous study, was especially strongly associated with ACPA-negative RF-positive RA (p = 5.0×10^−6^, OR: 4.97 for ACPA-negative RF-positive RA; p = 0.040, OR: 2.46 for ACPA-negative RF-negative RA).

We found that the similar associations were seen between the alleles/diplotypes and ACPA-negative RF-positive erosive RA and ACPA-negative RF-negative erosive RA (except for that between HLA-DRB1*12:01 and the ACPA-negative RF-negative subset), even though the number of patients was limited (Table S4).

### Comparison between ACPA-negative RF-positive RA and ACPA-negative RF-negative RA

To compare the usage of HLA-DRB1 allele between ACPA-negative RF-positive RA and ACPA-negative RF-negative RA, we directly compared the allele and diplotype frequencies between the two groups (Table 3). As expected, HLA-DRB1*09:01 and *04:05 showed significant differences in their frequencies between the two subsets (p = 0.0018 and 0.0034, respectively). The SE was more common in the ACPA-negative RF-positive RA patients (p = 0.0047), whereas DR14 was more prevalent in the ACPA-negative RF-negative RA patients (p = 0.028). The DR8 homozygote was more frequently seen in the ACPA-negative RF-negative RA patients than in the ACPA-negative RF-positive RA patients (p = 0.021). When we applied logistic regression analysis to the HLA-DRB1*09:01, *04:05, and HLA-DR14, their associations were revealed to be significant and do not depend on each other (p = 0.00067 and 0.00072, respectively, Table S5), except for that of DR14 (p = 0.30).

**Table 3:** Direct comparison of HLA-DRB1 allele frequency between ACPA-negative RF-positive RA and ACPA-negative RF-negative RA.

HLA-DRB1	ACPA(−)RF(+)RA Number of allele (%)	ACPA(−)RF(−)RA Number of allele (%)	*p*	OR (95%CI)
*09:01	169 (18.9%)	139 (13.7%)	0.0018	1.47 (1.15–1.88)
*04:05	153 (17.2%)	126 (12.4%)	0.0034	1.46 (1.13–1.89)
*08:02	24 (2.7%)	52 (5.1%)	0.0068	0.51 (0.31–0.84)
*14:06	8 (0.9%)	21 (2.1%)	0.037	0.43 (0.19–0.97)
SE	256 (28.7%)	234 (23.0%)	0.0047	1.35 (1.09–1.65)
DR14	78 (8.7%)	120 (11.8%)	0.028	0.72 (0.53–0.97)
DR8/DR8	6 (1.3%)	19 (3.7%)	0.021	0.35 (0.14–0.89)

### Comparison between ACPA-positive RF-positive RA and ACPA-positive RF-negative RA

**Figure 1:**
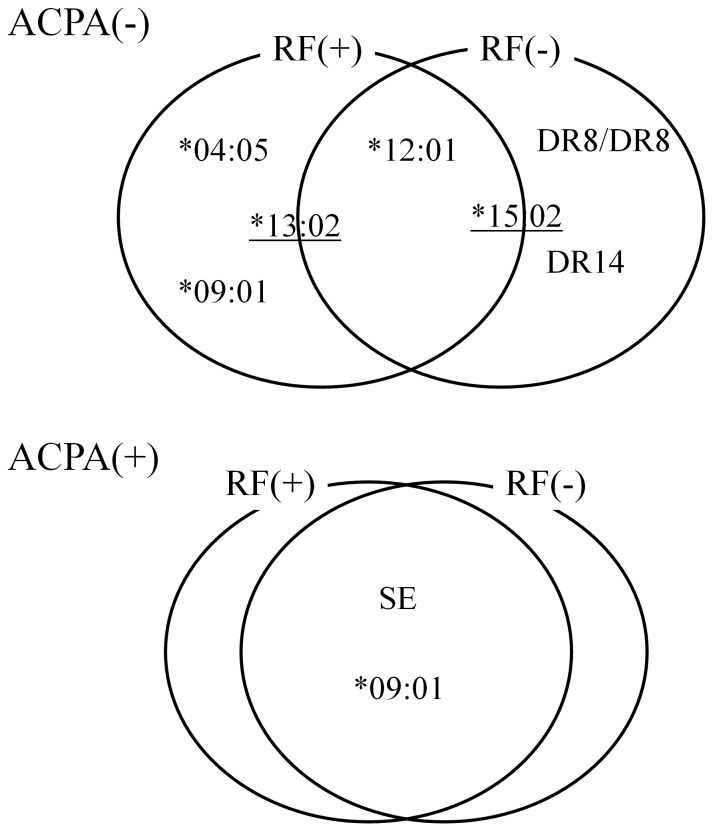
Summary of the HLA-DRB1 alleles associated with ACPA-negative RA and ACPA-positive RA. The relationships between the RF-positive and RF-negative subsets of ACPA-negative and ACPA-positive RA in terms of their associations with HLA-DRB1 alleles are illustrated. While the two subsets of ACPA-positive RA seem to share most associations with HLA-DRB1, the two ACPA-negative RA subsets possess specific alleles and HLA-DRB1 diplotypes. The underlined alleles are protective alleles.

Next, we analyzed whether these allele usage differences are also seen in ACPA-positive RA. We collected data about the HLA-DRB1 genotypes of 154 ACPA-positive RF-negative RA patients and 531 ACPA-positive RF-positive RA patients. As the SE and HLA-DRB1*09:01 were found to be associated with ACPA-positive RA, we analyzed the differences in the frequencies of these alleles [17]. In comparison with the healthy controls, SE and HLA-DRB1*09:01 were associated with a predisposition to ACPA-positive RF-positive RA as well as ACPA-positive RF-negative RA and displayed comparable odds ratios in logistic regression analysis (Table 4). No HLA-DRB1 alleles showed a strong specific association with a particular subset. When we directly compared the two subsets of ACPA-positive RA, no alleles displayed significant associations (Figure 1, Table S6). However, whether the two subsets of ACPA-positive RA share most of HLA-DRB1 susceptibility associations is inconclusive due to the small number of RF-negative subset.

**Table 4:** Logistic regression analysis of HLA-DRB1 alleles with ACPA-positive RF-positive RA and ACPA-positive RF-negative RA.

	ACPA(+)RF(+)RA		ACPA(+)RF(−)RA	
HLA-DRB1	*p* *	OR (95%CI)*	*p* *	OR (95%CI)*
SE	<2×10^−16^	3.21 (2.72–3.78)	<2×10^−16^	3.03 (2.33–3.94)
*09:01	2.4×10^−9^	1.83 (1.5–2.25)	0.0035	1.67 (1.17–2.37)

* *p*-values and odds ratios in logistic regression analysis using SE and HLA-DRB1*09:01.

## Discussion

In this study, we demonstrated that classifying Japanese ACPA-negative RA patients based on their RF positivity successfully divided them into two genetically different subsets, which displayed different associations with HLA-DRB1. We showed that HLA-DRB1*09:01 and *04:05, strong susceptibility alleles to ACPA-positive RA, were also associated with ACPA-negative RF-positive subset, and that DR14 and the DR8 homozygote were associated only with the ACPA-negative RF-negative subset (Figure 1). Since the titer of RF fluctuates along with disease activity much more than that of ACPA, we were very careful to take the maximum RF titer when multiple titers were available for a particular patient, in order to prevent the RF positive subset from being contaminated with RF negative RA patients. The Recent UK population study reported the association of SE with ACPA-negative RF-positive RA [16]. Our study not only confirmed this association in Japanese RA, but also showed that the association of SE with ACPA-negative RF-positive RA is mainly due to the effect of HLA-DRB1*04:05 and that HLA-DRB1*09:01, HLA-DR14, and homozygote of HLA-DR8 are specifically associated with subsets of ACPA-negative RA.

These above-mentioned association tendencies were observed in the first set and successfully replicated in the second set, indicating that we can avoid population stratification or sampling bias. The effect sizes (odds ratio) of the alleles were comparable in each cohort (Tables 1 and 2) and the associations in the combined analysis reached significant level, although the p-values in each set did not reach the significance level due to the limited number of samples they contained. These data indicate that our results are reliable, at least in Japanese populations, although further replication studies including other populations are favorable. In the current study, we used logistic regression analysis to confirm independency of associated alleles in each comparison. When we used relative predispositional effects (RPE) method [18] to stratify associated alleles, we obtained the similar results to those we obtained by logistic regression analysis (data not shown).

In our previous study [14], HLA-DRB1*09:01 was not significantly associated with ACPA-negative RA, in spite of the association it displayed in combination with HLA-DRB1*12:01. In the current study, we showed that HLA-DRB1*09:01 displayed a strong dose-dependent association with ACPA-negative RF-positive RA, but not with ACPA-negative RF-negative RA. These findings were confirmed by a direct comparison between the two subsets. A small study in Japan suggested that HLA-DRB1*09:01 is associated with ACPA-negative RA [15]. Our results suggest that their study mainly included ACPA-negative RF-positive RA patients. HLA-DRB1*09:01 was shown to reduce the ACPA titer in Japanese ACPA-positive RA patients [19]–[20]. Therefore, HLA-DRB1*09:01 might increase the titer of RF and decrease that of ACPA, although our study also showed that HLA-DRB1*09:01 is associated with ACPA-positive RF-negative RA.

HLA-DRB1*04:05, which is a major component of the SE in Asians [17], was shown to be significantly associated with ACPA-negative RA in our previous study. The current study showed that it is only associated with ACPA-negative RF-positive RA. This predisposition was also confirmed by direct comparison of the two subsets. As we could not detect a dosage effect of HLA-DRB1*04:05, its susceptibility effect might occur in a dominant manner. It is interesting that of the many SE alleles only HLA-DRB1*04:05 is associated with ACPA-negative RF-positive RA. This does not seem to be due to the relatively low frequencies of the other SE alleles (Table 1). Therefore, the common amino acid sequence that extends from the 70^th^ to the 74^th^ amino acid of the HLA-DRβ chain might not be important for the development of ACPA-negative RF-positive RA. As immunization of citrullinated peptide induced arthritis in HLA-DR4 transgenic mice [21] and citrullinated peptides were shown to have higher affinity to HLA-DR4 [22], high affinity of SE to citrullinated antigen is hypothesized to be the link between SE and RA development. Our findings may raise possibility of another mechanism of SE in developing arthritis.

It is quite interesting that HLA-DRB1*04:05 and *09:01, strongly associated alleles with ACPA-positive RA, are associated with ACPA-negative RF-positive RA. Although there are genetic similarities between ACPA-negative RF-positive RA and ACPA-positive RA, they should be considered to be different subsets as SE alleles other than HLA-DRB1*04:05 are not associated with ACPA-negative RF-positive RA and the HLA-DRB1*09:01 and *12:01 diplotype is strongly associated with ACPA-negative RF-positive RA.

When we analyzed the HLA-DR14 serotype, it showed a strong association with ACPA-negative RF-negative RA, largely due to HLA-DRB1*14:03 and *14:06. When we compared the frequency of DR14 in each ACPA-negative subset after stratifying the data according to the presence of HLA-DRB1*09:01 and *04:05, DR14 did not display a significant effect. In this sense, the specific association of DR14 with ACPA-negative RF-negative RA needs to be confirmed.

The HLA-DR8 homozygote displayed an association with ACPA-negative RA in our previous study [14]. The current study demonstrated that its association is specific to ACPA-negative RF-negative RA. As the number of HLA-DR8 homozygote is limited, further replication is necessary for this association. No association between the HLA-DR8 and 14 diplotype and susceptibility to ACPA-negative RF-negative RA was found (data not shown).

It is interesting that HLA-DR14 and HLA-DR8, associated serotype with ACPA-negative RF-negative RA, were reported association with psoriatic arthritis [23]. HLA-DR14 is often linked with HLA-Cw*06, susceptibility serotype to psoriasis arthritis in European [24]. HLA-Cw*06 is rare in Japanese (<1%) and the strong association between HLA-Cw*06 and HLA-DR14 is not observed in Japan (<10%). While psoriatic arthritis is not reported to be associated with these serotypes in Japan, association between these serotypes and arthritis is interesting.

It could be argued that ACPA-negative RA includes some non-RA arthritic diseases such as psoriasis, seronegative spondyloarthropathy and other collagen vascular diseases. Thus, we analyzed the associations between the above-mentioned alleles and diplotypes with ACPA-negative RA displaying bone erosion to examine whether the same association patterns were present in this strictly defined cohort. The typical bone erosions of RA are rarely seen in other arthritic disorders. As a result, we found the same associations. Therefore, we are convinced that our findings were not caused by the contamination of our study population by patients with other diseases. Since RF sometimes normalizes after treatment, the RF-negative RA patients whose RF titers were not measured at multiple points might not have been RF-negative. So, we re-analyzed our data by excluding the RA patients for whom consecutive RF titers were not available. As a result, we found the same tendency of associations for each allele and diplotype in each subset (data not shown), indicating that these subsets are stable.

Analysis using ACPA-positive RF-positive RA and ACPA-positive RF-negative RA patients compared with healthy controls did not result in distinct differences in HLA-DRB1 association. The SE is associated with both ACPA-positive RF-positive and RF-negative RA. HLA-DRB1*09:01 was found to be associated with both subsets after stratifying the patients according to their SE alleles. We also did not detect an association between HLA-DR14 or the HLA-DR8 homozygote and either subset. While 154 ACPA-positive RF-negative RA patients in our study are too small in number to detect the difference in HLA-DRB1 alleles with weak effect size between the two ACPA-positive subsets, these results suggest that there are no big differences in the HLA usage of the two subsets in ACPA-positive RA. To confirm our results and to detect possible different frequency of other HLA-DRB1 alleles in the two ACPA-positive subsets, replication study is necessary.

In the current study, we performed multiple comparisons in each subset and between subsets. The associations should be evaluated in the combined analysis with significant level corrected by Bonferronii’s method and independency of each association should be evaluated by logistic regression analysis or RPE method. In this sense, p-values around cut-off level in the combined analysis should be taken with caution and the associations should be confirmed by independent study.

We have shown that ACPA-negative RA includes two genetically distinct subsets in Japanese population: RF-positive and RF-negative RA. This is the first report in Asians to show that these subsets are genetically distinct. We have to clarify the clinical difference between these two subsets. We also have to clarify whether non-HLA genes display different associations with each subset. So far, many genome wide association studies (GWAS) of RA and ACPA-positive RA have been performed, and more than twenty genes or loci have been shown to be susceptibility loci [25]–[38]. However, no GWAS studies have detected susceptibility genes for ACPA-negative RA with genome-wide significance [39]. This is probably due to the relatively small number of patients studied, but it might be overcome by stratifying ACPA-negative RA patients into RF-positive and RF-negative subsets. Since RA susceptibility genes usually cross ethnic boundaries [40], global collaboration might result in a fruitful dissection of these minor subsets.

## Materials and Methods

### Ethics Statement

This study was approved by the local ethical committees at each institution, namely, Kyoto University Graduate School and Faculty of Medicine, Ethics Committee, Tokyo Women’s Medical University Genome Ethics Committee, and the ethics committee of RIKEN, and written informed consent was obtained from all patients.

### Study Subjects

DNA samples were collected from ACPA-negative RA patients at Kyoto University Hospital, Tokyo Women’s Medical University [41], and RIKEN with the support of BioBank Japan. All patients were Japanese and had been diagnosed by rheumatologists according to the 1987 American College of Rheumatology revised criteria for RA [42]. The control DNA samples were collected at Aichi Cancer Center Hospital, the DNA banks of the Pharma SNP Consortium [43], and HLA laboratory. A more detailed description of the collection procedure was given in a previous study [14]. We performed association studies using similar study design of the two collections to our previous study; namely, collection 1 for 456 ACPA-negative RA and 1508 healthy subjects, and collection 2 for 501 ACPA-negative RA and 500 healthy people. RF data were available for 453 out of 456 cases in collection 1 and all of 501 cases in collection 2. 179 patients were RF-positive in collection 1 and 267 patients were RF-positive in collection 2. We also collected DNA samples from 531 ACPA-positive RF-positive RA patients at Kyoto University Hospital and 154 ACPA-positive RF-negative RA patients at Kyoto University and Tokyo Women’s Medical University.

### ACPA Detection

The MESACUP CCP ELISA kit (Medical and Biological Laboratories Co., Ltd, Nagoya, Japan) was used to detect 2^nd^ generation ACPA in each RA patient, according to the manufacturer’s instructions. A cut-off value of 4.5 U/ml was used to define ACPA positivity.

### RF Detection

The serum RF concentrations of samples in collection 1 were quantified using a latex agglutination turbidimetric immunoassay. An ELISA assay was used to determine the RF levels of samples in collection 2. When multiple values for RF had been obtained at different visits, we used the maximum RF value for each patient. The cut off values of each detection kit in each hospital were employed.

### HLA-DRB1 Genotyping

The HLA-DRB1 typing methods were previously described [14]. Briefly, the WAKFlow system or the AlleleSEQR HLA-DRB1 typing kit (Abbott, Tokyo, Japan) was used for the HLA-DRB1 typing. The following HLA-DRB1 alleles were classified as belonging to the SE: DRB1*01:01, *01:02, *04:01, *04:04, *04:05, *04:08, *04:10, *04:13, *04:16, *10:01, *14:02, and *14:06.

### Statistical Analysis

The frequency of each allele or diplotype was compared among the ACPA-negative RF-positive RA, ACPA-negative RF-negative RA patients, and the healthy controls in each set and combined set using the chi-square test or Fisher’s exact test. The same analyses were performed in ACPA-positive RA patients classified according to their RF possession. Ninety-five percent confidence intervals (CI) for the OR were also calculated. Logistic regression analysis was used to evaluate the effects of each allele by adjusting for the influence of strongly-associated alleles. Single alleles were regarded as significant when they showed p-values of less than 0.0026 in a combined study, which is obtained by Bonferroni’s correction. For diplotype analyses, we regarded 0.025 as the cut off level for significance because we performed just two tests. All statistical analyses were performed using the R statistic system (http://www.R-project.org) or SPSS (version 18).

## Supporting Information

Figure S1 Dosage effects of HLA-DRB1*04:05 and *09:01 alleles on ACPA-negative RF-positive RA susceptibility. Each column represents the odds ratio for developing ACPA-negative RF-positive RA associated with possessing one (red column) or two (green column) alleles of HLA-DRB1*04:05 or *09:01.
(TIF)

Table S1 Logistic regression analysis of associated alleles with ACPA-negative RF-positive RA. *p-values and odds ratios in logistic regression analysis using the six alleles listed above.
(DOC)

Table S2 Association between HLA-DR14 and ACPA-negative RF-negative RA.
(DOC)

Table S3 Logistic regression analysis of associated alleles with ACPA-negative RF-negative RA. *p-values and odds ratios in logistic regression analysis using HLA-DR14 and three HLA-DRB1 alleles listed above.
(DOC)

Table S4 Association of HLA-DRB1 with ACPA-negative RA erosive subsets. ^a)^Total allele number is 268. ^b)^Total allele number is 212.
(DOC)

Table S5 Logistic regression analysis of assoicated alleles with ACPA-negative RF-positive RA, compared with ACPA-negative RF-negative RA. *p-values and odds ratios in logistic regression analysis using HLA-DRB1*09:01, *04:05, and HLA-DR14. ^a)^HLA-DRB1 alleles which showed p<0.05 in Table 3 were used for analysis.
(DOC)

Table S6 Comparison between ACPA-positive RF-positive RA and ACPA-positive RF-negative RA. ^a)^ Alleles with frequency more than 1% in any groups are shown.
(DOC)
