# Supplementary figures and images for: ACPA-Negative RA Consists of Two Genetically Distinct Subsets Based on RF Positivity in Japanese
Source: PLoS One. 2012 Jul 6;7(7):e40067. doi: 10.1371/journal.pone.0040067 (PMC3391228; doi:10.1371/journal.pone.0040067)

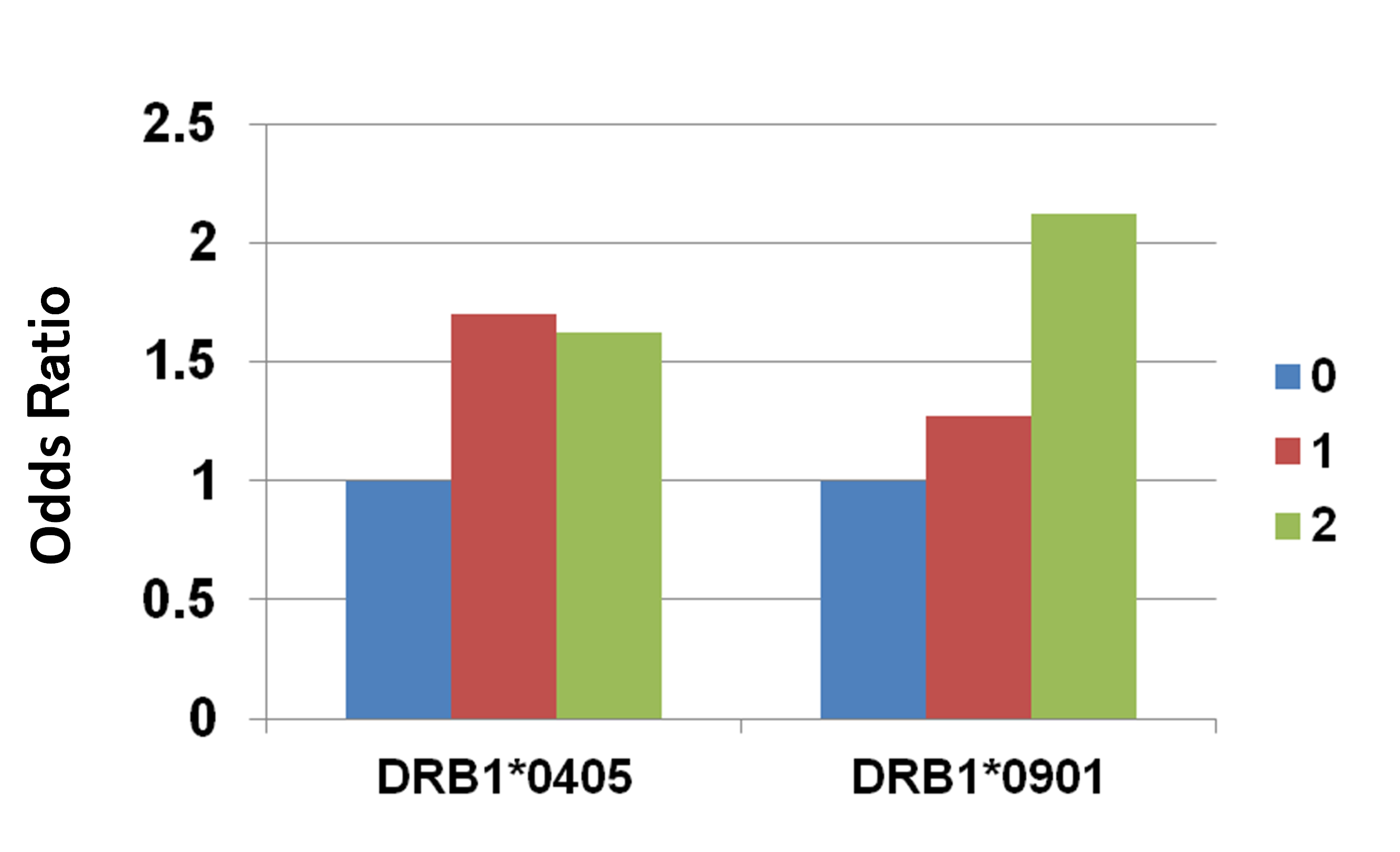

Supplement: Figure S1 — Dosage effects of HLA-DRB1*04:05 and *09:01 alleles on ACPA-negative RF-positive RA susceptibility. Each column represents the odds ratio for developing ACPA-negative RF-positive RA associated with possessing one (red column) or two (green column) alleles of HLA-DRB1*04:05 or *09:01. (TIF) [file pone.0040067.s001.tif]
