# Supplementary material for: ACPA-Negative RA Consists of Two Genetically Distinct Subsets Based on RF Positivity in Japanese
Source: PLoS One. 2012 Jul 6;7(7):e40067. doi: 10.1371/journal.pone.0040067 (PMC3391228; doi:10.1371/journal.pone.0040067)
Supplement: Table S1 — Logistic regression analysis of associated alleles with ACPA-negative RF-positive RA. *p-values and odds ratios in logistic regression analysis using the six alleles listed above. (DOC) [file pone.0040067.s002.doc]

| HLA-DRB1 | *p** | OR(95%CI)* |
| --- | --- | --- |
| *04:05 | 7.8x10^-7^ | 1.72 (1.38-2.14) |
| *09:01 | 0.00010 | 1.49 (1.21-1.83) |
| *12:01 | 0.00021 | 1.97 (1.37-2.83) |
| *13:02 | 0.012 | 0.64 (0.45-0.91) |
| *15:02 | 0.19 | 0.84 (0.64-1.1) |
| *14:03 | 0.0066 | 2.11 (1.22-3.64) |
