# Supplementary material for: ACPA-Negative RA Consists of Two Genetically Distinct Subsets Based on RF Positivity in Japanese
Source: PLoS One. 2012 Jul 6;7(7):e40067. doi: 10.1371/journal.pone.0040067 (PMC3391228; doi:10.1371/journal.pone.0040067)
Supplement: Table S2 — Association between HLA-DR14 and ACPA-negative RF-negative RA. (DOC) [file pone.0040067.s003.doc]

|  | Collection 1 | | | |  | Collection 2 | | | |  | Combined analysis | | | |
| --- | --- | --- | --- | --- | --- | --- | --- | --- | --- | --- | --- | --- | --- | --- |
| HLA-DRB1 | ACPA(-)RF(-)RA | Control | *p* | OR (95%CI) |  | ACPA(-)RF(-)RA | Control | *p* | OR (95%CI) |  | ACPA(-)RF(-)RA | Control | *p* | OR (95%CI) |
| DR14 |  |  |  |  |  |  |  |  |  |  |  |  |  |  |
| 14:03 | 12 (2.2%) | 39 (1.3%) | 0.10 | 1.72 (0.89-3.3) |  | 10 (2.1%) | 14 (1.4%) | 0.30 | 1.54 (0.68-3.49) |  | 22 (2.2%) | 53 (1.3%) | 0.047 | 1.66 (1.00-2.73) |
| 14:06 | 13 (2.4%) | 37 (1.2%) | 0.036 | 1.96 (1.03-3.71) |  | 8 (1.7%) | 9 (0.9%) | 0.18 | 1.91 (0.73-5.00) |  | 21 (2.1%) | 46 (1.1%) | 0.022 | 1.82 (1.08-3.07) |
| other DR14 | 44 (8.1%) | 177 (5.9%) | 0.054 | 1.40 (0.99-1.97) |  | 33 (7.1%) | 50 (5.0%) | 0.11 | 1.44 (0.92-2.27) |  | 77 (7.6%) | 227 (5.7%) | 0.021 | 1.37 (1.05-1.79) |
