# Supplementary material for: ACPA-Negative RA Consists of Two Genetically Distinct Subsets Based on RF Positivity in Japanese
Source: PLoS One. 2012 Jul 6;7(7):e40067. doi: 10.1371/journal.pone.0040067 (PMC3391228; doi:10.1371/journal.pone.0040067)
Supplement: Table S3 — Logistic regression analysis of associated alleles with ACPA-negative RF-negative RA. *p-values and odds ratios in logistic regression analysis using HLA-DR14 and three HLA-DRB1 alleles listed above. (DOC) [file pone.0040067.s004.doc]

| HLA-DRB1 | *p** | OR(95%CI)* |
| --- | --- | --- |
| DR14 | 0.00069 | 1.48 (1.18-1.87) |
| *12:01 | 0.0015 | 1.70 (1.22-2.38) |
| *15:02 | 0.0058 | 0.71 (0.55-0.91) |
| *13:02 | 0.074 | 0.77 (0.58-1.03) |
