# Supplementary material for: ACPA-Negative RA Consists of Two Genetically Distinct Subsets Based on RF Positivity in Japanese
Source: PLoS One. 2012 Jul 6;7(7):e40067. doi: 10.1371/journal.pone.0040067 (PMC3391228; doi:10.1371/journal.pone.0040067)
Supplement: Table S4 — Association of HLA-DRB1 with ACPA-negative RA erosive subsets. a)Total allele number is 268. b)Total allele number is 212. (DOC) [file pone.0040067.s005.doc]

|  | Control |  | ACPA(-)RF(+)  erosive RA^a)^ | | |  | ACPA(-)RF(-)  erosive RA^b)^ | | |
| --- | --- | --- | --- | --- | --- | --- | --- | --- | --- |
| HLA-DRB1 | Number of  alleles (%) |  | Number of  alleles (%) | *p* | OR (95%CI) |  | Number of  alleles (%) | *p* | OR (95%CI) |
| *04:05 | 469 (11.7%) |  | 52 (19.4%) | 0.00018 | 1.82 (1.33-2.50) |  | 30 (14.2%) | 0.28 | 1.25 (0.84-1.86) |
| *09:01 | 586 (14.6%) |  | 56 (20.9%) | 0.0051 | 1.55 (1.14-2.10) |  | 32 (15.1%) | 0.84 | 1.04 (0.71-1.53) |
| *12:01 | 128 (3.2%) |  | 13 (4.9%) | 0.14 | 1.55 (0.86-2.78) |  | 7 (3.3%) | 0.93 | 1.04 (0.48-2.25) |
| *13:02 | 325 (8.1%) |  | 13 (4.9%) | 0.057 | 0.58 (0.33-1.02) |  | 9 (4.2%) | 0.043 | 0.50 (0.26-0.99) |
| *14:03 | 53 (1.3%) |  | 4 (1.5%) | 0.81 | 1.13 (0.41-3.15) |  | 7 (3.3%) | 0.017 | 2.55 (1.15-5.69) |
| *15:02 | 482 (12.0%) |  | 22 (8.2%) | 0.062 | 0.66 (0.42-1.02) |  | 15 (7.1%) | 0.030 | 0.56 (0.33-0.95) |
|  |  |  |  |  |  |  |  |  |  |
| SE | 910 (22.7%) |  | 88 (32.8%) | 0.00014 | 1.67 (1.28-2.18) |  | 48 (22.6%) | 1.00 | 1.00 (0.72-1.39) |
| DR14 | 326 (8.1%) |  | 18 (6.7%) | 0.41 | 0.81 (0.50-1.33) |  | 22 (10.4%) | 0.24 | 1.31 (0.83-2.07) |
|  |  |  |  |  |  |  |  |  |  |
| DR8/DR8 | 25 (1.2%) |  | 2 (1.5%) | 0.68 | 1.20 (0.14-4.91) |  | 4 (3.8%) | 0.054 | 3.11 (0.77-9.24) |
| *09:01/*12:01 | 13 (0.6%) |  | 3 (2.2%) | 0.074 | 3.51 (0.63-13.00) |  | 1 (0.9%) | 0.51 | 1.46 (0.034-9.91) |
