# Supplementary material for: ACPA-Negative RA Consists of Two Genetically Distinct Subsets Based on RF Positivity in Japanese
Source: PLoS One. 2012 Jul 6;7(7):e40067. doi: 10.1371/journal.pone.0040067 (PMC3391228; doi:10.1371/journal.pone.0040067)
Supplement: Table S5 — Logistic regression analysis of assoicated alleles with ACPA-negative RF-positive RA, compared with ACPA-negative RF-negative RA. *p-values and odds ratios in logistic regression analysis using HLA-DRB1*09:01, *04:05, and HLA-DR14. a)HLA-DRB1 alleles which showed p<0.05 in Table 3 were used for analysis. (DOC) [file pone.0040067.s006.doc]

| HLA-DRB1^a)^ | *p** | OR(95%CI)* |
| --- | --- | --- |
| *09:01 | 0.00067 | 1.55 (1.20-2.01) |
| *04:05 | 0.00072 | 1.60 (1.21-2.11) |
| DR14 | 0.30 | 0.84 (0.61-1.17) |
