# Supplementary material for: ACPA-Negative RA Consists of Two Genetically Distinct Subsets Based on RF Positivity in Japanese
Source: PLoS One. 2012 Jul 6;7(7):e40067. doi: 10.1371/journal.pone.0040067 (PMC3391228; doi:10.1371/journal.pone.0040067)
Supplement: Table S6 — Comparison between ACPA-positive RF-positive RA and ACPA-positive RF-negative RA. a) Alleles with frequency more than 1% in any groups are shown. (DOC) [file pone.0040067.s007.doc]

| HLA-DRB1^a)^ | ACPA(+)RF(+)RA | ACPA(+)RF(-)RA | *P* | OR (95%CI) |
| --- | --- | --- | --- | --- |
| *01:01 | 75 (7.1%) | 22 (7.1%) | 0.96 | 0.99 (0.6-1.62) |
| *04:01 | 31 (2.9%) | 8 (2.6%) | 0.77 | 1.13 (0.51-2.48) |
| *04:03 | 18 (1.7%) | 7 (2.3%) | 0.50 | 0.74 (0.31-1.79) |
| *04:05 | 323 (30.4%) | 85 (27.6%) | 0.34 | 1.15 (0.86-1.52) |
| *04:06 | 20 (1.9%) | 7 (2.3%) | 0.67 | 0.83 (0.35-1.97) |
| *04:10 | 20 (1.9%) | 11 (3.6%) | 0.079 | 0.52 (0.25-1.09) |
| *08:02 | 18 (1.7%) | 12 (3.9%) | 0.020 | 0.43 (0.2-0.89) |
| *08:03 | 53 (5.0%) | 7 (2.3%) | 0.040 | 2.26 (1.02-5.02) |
| *09:01 | 176 (16.6%) | 48 (15.6%) | 0.68 | 1.08 (0.76-1.52) |
| *10:01 | 10 (0.9%) | 6 (1.9%) | 0.15 | 0.48 (0.17-1.33) |
| *11:01 | 23 (2.2%) | 3 (1.0%) | 0.18 | 2.25 (0.67-7.55) |
| *12:01 | 28 (2.6%) | 7 (2.3%) | 0.72 | 1.16 (0.5-2.69) |
| *12:02 | 15 (1.4%) | 5 (1.6%) | 0.79 | 0.87 (0.31-2.41) |
| *13:02 | 31 (2.9%) | 11 (3.6%) | 0.56 | 0.81 (0.4-1.63) |
| *14:01 | 23 (2.2%) | 8 (2.6%) | 0.65 | 0.83 (0.37-1.87) |
| *14:03 | 7 (0.7%) | 6 (1.9%) | 0.040 | 0.33 (0.11-1) |
| *14:05 | 11 (1.0%) | 4 (1.3%) | 0.70 | 0.8 (0.25-2.52) |
| *14:06 | 9 (0.8%) | 5 (1.6%) | 0.23 | 0.52 (0.17-1.56) |
| *15:01 | 55 (5.2%) | 19 (6.2%) | 0.50 | 0.83 (0.49-1.42) |
| *15:02 | 89 (8.4%) | 15 (4.9%) | 0.041 | 1.79 (1.02-3.14) |
| *16:02 | 13 (1.2%) | 5 (1.6%) | 0.59 | 0.75 (0.27-2.12) |
|  |  |  |  |  |
| SE | 474 (44.6%) | 138 (44.8%) | 0.96 | 0.99 (0.77-1.28) |
